# Effect of preoperative jaundice on long-term prognosis of gallbladder carcinoma with radical resection

**DOI:** 10.1186/s12957-020-02015-2

**Published:** 2020-09-05

**Authors:** Xin-wei Yang, Jun-yi Chen, Zhi-jian Wen, Yu-long Li, Fei-yu Wang, Liang Li, Jue Yang, Ping-hua Yang, Bao-hua Zhang, Feng Shen

**Affiliations:** 1 Eastern Hepatobiliary Surgery Hospital, Second Military Medical University, Changhai Road 225, Shanghai, 200438 China; 2 Department of General Surgery, The Fourth People’s Hospital of Shanghai, North Sichuang Road 1878, Shanghai, 200081 Shanghai China; 3 Department of General Surgery, No.73 Army Hospital of PLA, Xiamen University, Xiamen, China; 4 Department of General Surgery, Jiangdu People’s Hospital of Yangzhou City, Jiangsu Province, No. 9 Dongfanghong East Road, Yangzhou, China

**Keywords:** Jaundice, Gallbladder carcinoma, R0, Long-term prognosis, pT stage

## Abstract

**Purposes:**

This study was designed to evaluate the effect of preoperative jaundice on long-term prognosis of gallbladder carcinoma (GBC) after radical resection (R0).

**Methods:**

A total of 267 GBC patients who underwent R0 resection from January 2004 to December 2014 were enrolled, including 54 patients with preoperative jaundice and 213 patients without jaundice. The clinicopathological parameters between the two groups were compared, and the correlation between preoperative jaundice and the long-term prognosis was furtherly analyzed.

**Results:**

Unilateral and multivariate analyses of 267 GBC patients showed that the depth of tumor invasion (pT stage), lymphatic metastasis, and hepatic invasion were independent prognostic factors. The univariate and multivariate analysis of 54 GBC patients with preoperative jaundice showed that only pT stage was an independent factor for prognosis. Furthermore, the intraoperative blood transfusion and pT stage were significant different between long-term survival (survive for more than 3 years) and those who died within 3 years (*P* < 0.05).

**Conclusion:**

Preoperative jaundice was not the independent factor resulting in the poor long-term prognosis of gallbladder carcinoma after R0 resection. The pT stage was the only long-term prognostic factor in all GBC patients regardless of preoperative jaundice.

## Introduction

Gallbladder carcinoma (GBC) that has invaded the submucosa without lymph node metastasis may have a favorable prognosis after surgical resection [1]. However, GBC has a tendency to invade the surrounding organs, especially to the hilar and hepatoduodenal ligaments. This usually leads to obstructive jaundice, which usually indicates that the disease is in advanced stage and cannot be surgically resected [2].

Previous reports have suggested that GBC patients with preoperative jaundice were significantly associated with poor prognosis [3–7]. In addition, some recent studies also have found that preoperative jaundice or extrahepatic bile duct invasion were independent predictors of poor prognosis [8, 9]. Even so, some scholars supported surgical resection in such advanced patients [10, 11]. Our previous study [12] has found that the jaundiced patients have lower survival rates than the non-jaundiced patients. The multivariate analysis showed that preoperative jaundice was not a significant risk factor of poor outcome in GBC patients who underwent surgical resection with curative intent (R0 and R1 resections) [12]. However, the role of preoperative jaundice in the prognosis evaluation of GBC patients after R0 resection has never been reported. The aim of this study was to evaluate the long-term prognostic value of preoperative jaundice in GBC patients after R0 resection.

## Materials and methods

### Patients

A prospectively maintained hepatobiliary surgery database at the Eastern Hepatobiliary Hospital was reviewed for all patients with a diagnosis of GBC who underwent surgical resection with curative intent between January 2004 and December 2014. Permission from the Second Military Medical University’s Institutional Review Board was obtained prior to data review. Written informed consent was obtained from all patients for surgical treatment and pathological examinations according to the institutional guidelines.

Preoperative jaundice was defined as the elevated total bilirubin level (> 3.0 mg/dl). Resection completeness was classified into R0 microscopically margin-negative resection, R1 microscopically positive margin, and R2 macroscopic residuals on surgical margins. R0 was considered to be radical resection. During the operation, 10 patients underwent rapid freezing to confirm the negative margin, which was confirmed by the final pathological results. All patients who had undergone either R1 or R2 surgery were excluded from the analysis. All surgeries were performed by a single treatment team. Stage grouping was performed according to the pTNM classification system of UICC, 8th edition [13].

### Surgical procedures

All patients were given preoperative imaging to assess the accurate range of tumor invasion, before they were scheduled for surgery. In order to reduce the risk of postoperative liver failure, preoperative portal vein embolization (PVE) was performed in patients with more than 60% of hepatic parenchymal resection [6]. Hepatectomy was scheduled 2–3 weeks after PVE when liver hypertrophy had been confirmed by volumetry, and the serum bilirubin level was lower than 6 mg/dl. Two patients underwent hemihepatic PVE prior to extended right hemihepatectomy.

All patients underwent en bloc dissection of regional lymph nodes (lymph nodes along the hepatoduodenal ligament and common hepatic artery and behind the pancreatic head). Hepatectomies were carried out in all 276 patients. When the adjacent organ was found to be invaded, en bloc resection was performed simultaneously, such as pancreaticoduodenectomy, partial gastrectomy, partial duodenal resection, and colon resection. Vascular resection (hepatic artery and/or portal vein) was carried out when R0 resection was expected.

### Statistical analysis

Overall survival was measured from the day of operation to death, including the death caused by cancer or other factors, or to the last day of follow-up. All patients were followed up for more than 5 years unless the patients died within 5 years. The average follow-up time was 76.7 months. The comparison between the two groups was done by Student’s *t* test for parametric data and the Mann–Whitney *U* test for non-parametric data. The Chi-square test was used for categorical data. Survival curves were estimated with the Kaplan–Meier method and compared by the log-rank test. Cox regression analysis was carried out to determine which factor was the best prognostic determinant. *P* value < 0.05 was considered statistically significant. Calculations were done by SPSS Version 17.0 for Windows (SPSS, Inc., Chicago, IL, USA).

## Results

### Demographic data

In this 10-year study, 536 patients with gallbladder carcinoma underwent surgery, of which 267 patients underwent radical resection (R0 resection, 49.8%), 183 underwent R1 resection, and 86 underwent R2 resection. Among the 267 GBC patients who underwent R0 resection, 105 were male and 162 were female, including 54 patients with preoperative jaundice. The average age was 58.6 years (range 23–83 years).

### Preoperative management

All 54 GBC patients with preoperative jaundice routinely underwent extrahepatic bile duct resection. The mean total bilirubin in 54 GBC patients with preoperative jaundice was 11.4 mg/dl (range 2.89–33.01 mg/dl). Of the 54 patients, 34 patients (61.1%) underwent preoperative biliary drainage according to liver function and the expected range of liver resection. Eighteen cases were treated with percutaneous transhepatic biliary puncture (PTBD), and 16 cases were treated with endoscopic biliary drainage (EBD). Among the 34 patients who underwent preoperative biliary drainage, the mean total bilirubin at admission was 15.6 mg/dl (range 7.79–33.01 mg/dl), and decreased to 4.7 mg/dl (range 3.2–5.8 mg/dl) before surgery. Four patients developed cholangitis, and two patients developed hemorrhage associated with preoperative biliary drainage, who were treated without sequela after conservative treatment.

### Comparison between GBC patients with and without preoperative jaundice who underwent R0 resection (Table 1)

There were no significant differences in age, associated gallbladder disease, histological differentiation, and hepatic invasion between patients with and without preoperative jaundice. Male patients were more common in the preoperative jaundice group. A more advanced pT stage was associated with preoperative jaundice, which suggested a more serious local tumor invasion. The extended hepatectomy was more common in the preoperative jaundice group. More intraoperative bleeding and operative time were found in the preoperative jaundice group (*P* < 0.001 and P = 0.001), which suggested a wider range of lesion resection performed. As a result, the average postoperative hospital stay of GBC patients with preoperative jaundice was 18.3 days (range 4–85 days), which was longer than those without preoperative jaundice (*P* < 0.001).

**Table 1:** Demographic data of jaundiced (*n* = 54) and non-jaundiced GBC patients (*n* = 213)

	Jaundiced, *n*	Non-jaundiced, *n*	*P* value
Male gender	30	75	0.006
Mean age (range)	58.04 ± 10.87 (35–80)	59.21 ± 10.60 (23–83)	0.478
Postoperative hospital stay	18.31 ± 12.91 (4–85)	10.92 ± 5.66 (5–51)	< 0.001
Associated gallbladder disease			0.285
Gallstones	24 (44.4%)	116 (54.5%)	
Gallbladder polyp	1 (1.9%)	2 (0.9%)	
Nil	29 (53.7%)	95 (44.6%)	
Tumor location			< 0.001
Gallbladder neck	38 (70.4)	29 (13.6)	
Gallbladder body + fundus	16 (29.6)	184 (86.4)	
Histologic type			0.107
Moderately + well-differentiated	51 (94.4)	182 (85.4)	
Poorly differentiated	3 (5.6)	31 (14.6)	
Extent of liver resection			< 0.001
Major hepatectomy (> 3 segments)	4 (7.4%)	1 (0.6%)	
Anatomical segments IV–V	20 (37.0%)	146 (68.5%)	
Gallbladder bed	30 (55.6%)	66 (30.9%)	
Extrahepatic bile duct resection	54 (100.0%)	20(9.4%)	< 0.001
Combined resection of adjacent organs	9 (16.7%)	9 (4.2%)	0.003
Hepatic invasion	29 (53.7%)	100 (46.9%)	0.446
Lymph node metastasis	39 (72.2%)	94 (44.1%)	< 0.001
Vascular invasion	5(9.3%)	0 (0%)	< 0.001
pT			0.004
pT2	0 (0%)	25 (11.7%)	
pT3	45 (83.3%)	174 (81.7%)	
pT4	9(16.7%)	14 (6.6%)	
Intraoperative bleeding (ml)	656.48 ± 532.97 (200–3200)	329.67 ± 257.13 (200–1800)	< 0.001
Operative time (min)	296.02 ± 76.91 (100–470)	211.88 ± 80.10 (100–400)	< 0.001
Mortality (number of patients)	2 (3.7%)	1 (0.5%)	0.105
Morbidity (need invasive treatment)	15 (27.8%)	13 (6.1%)	< 0.001

Note that adjacent organs include the pancreas, duodenum, stomach, and/or colon other than the liver and extrahepatic bile duct.

In terms of tumor location, the most commonly occurring place was at the neck of the gallbladder in the preoperative jaundice group. There was no significant difference in the incidence of hepatic invasion between the two groups, although the incidence of lymphatic metastasis was significantly higher in the preoperative jaundice group. There was no significant difference in mortality between GBC patients with and without preoperative jaundice (*P* = 0.105). Morbidity was significantly higher in patients with preoperative jaundice than without preoperative jaundice (27.8% vs. 6.1%, *P* < 0.001).

### Survival and risk factors in all 267 GBC patients who underwent R0 resection (Tables 2 and 3)

The overall 3-year and 5-year survival rates of 267 patients were 35.2% and 23.7%, respectively. The mean survival time was 36 months. According to the presence or absence of preoperative jaundice, the prognosis of 267 patients were analyzed. The 3-year survival rate and mean survival time were 40.9% and 40.8 months in 213 patients without preoperative jaundice, and 13.0% and 18.0 months in 54 patients with preoperative jaundice, respectively. The survival rate of patients with preoperative jaundice group was significantly worse than those in the group without jaundice (*P* < 0.001).

**Table 2:** Univariate analysis of 14 variables related to survival of GBC patients who underwent curative resection (*n* = 267)

Variable	Cutoff level	Number	Survival rates (%)		*P* value
			3 years	5 years	
Age (years)					0.019
	< 60	133	42.2	32.0	
	≥ 60	134	28.2	15.3	
Sex					0.158
	Male	105	29.8	20.2	
	Female	162	38.7	25.7	
Jaundice					< 0.001
	Present	54	13.0	1.9	
	Absent	213	40.9	29.8	
Associated disease					0.654
	Present	143	36.0	25.2	
	Absent	124	34.5	22.3	
Tumor location					0.002
	Gallbladder neck	67	20.3	9.4	
	Gallbladder body/fundus	200	40.2	28.9	
pT (TNM)					< 0.001
	pT1 and 2	25	83.8	78.6	
	pT3 and 4	242	30.2	18.0	
Lymph node metastasis					< 0.001
	Negative	134	52.2	38.9	
	Positive	133	18.1	9.3	
Histologic differentiation					0.049
	Well/moderate	224	27.4	17.4	
	Poor	43	36.7	24.9	
Hepatic invasion					< 0.001
	Present	129	20.6	11.1	
	Absent	138	48.8	35.6	
CRAO					< 0.001
	Present	18	5.6	0.0	
	Absent	249	36.9	25.0	
Combined portal vein/hepatic artery resection					0.027
	Present	5	0.0	0.0	
	Absent	262	35.8	24.1	
Intraoperative blood infusion					0.006
	Present	44	25.0	10.9	
	Absent	223	37.1	26.5	
Morbidity					0.004
	Conservative treatment	239	37.6	25.1	
	Need invasive treatment	28	14.3	5.4	
Extrahepatic bile duct resection					< 0.001
	Present	74	18.3	8.4	
	Absent	193	41.7	30.0

**Table 3:** Results of multivariate analysis

Variable	Regression coefficient	Standard error	*P* value	Relative risk	95% CI
Age	0.227	0.148	0.125	1.255	0.939-1.679
Jaundice	0.375	0.321	0.243	1.455	0.776-2.731
Tumor location	-0.390	0.207	0.060	0.677	0.451-1.016
pT	0.796	0.360	0.027	2.217	1.095-4.491
Lymph node metastasis	0.537	0.158	0.001	1.711	1.255-2.331
Hepatic invasion	0.677	0.157	0.000	1.968	1.446-2.678
CRAO	0.160	0.296	0.588	1.174	0.657-2.096
Combined PV/HA resection	0.536	0.497	0.280	1.710	0.646-4.528
Intraoperative blood infusion	-0.194	0.212	0.360	0.824	0.543-1.248
Extrahepatic bile duct resection	-0.147	0.275	0.592	0.863	0.503-1.480
Histologic differentiation	-0.360	0.200	0.071	0.697	0.472-1.031
Morbidity (invasive treatment)	0.078	0.253	0.759	1.081	0.658-1.775

Univariate and multivariate analyses were given to determine the significant factors that affected long-term survival in 267 GBC patients who underwent R0 resection. In the univariate analysis, the significant prognostic factors include the depth of tumor invasion (pT stage), age, preoperative jaundice, tumor location, lymph node metastasis, hepatic invasion, combined resection of adjacent organs (CRAO), portal vein/hepatic artery (HA/PV) resection, intraoperative blood transfusion, extrahepatic bile duct resection, treatment of complications, and histologic differentiation (Table 2). In the multivariate analysis, only pT stage, lymph node metastasis, and hepatic invasion were independent factors (Table 3).

### Risk factors in 54 GBC patients with preoperative jaundice who underwent R0 resection (Table 4)

Univariate and multivariate analysis were given to identify risk factors in 54 GBC patients with preoperative jaundice who underwent R0 resection. In the univariate analysis, pT stage, tumor location, and histologic differentiation were identified as significant prognostic factors. In the multivariate analysis, only advanced pT stage was an independent risk factor for poor prognosis.

**Table 4:** Univariate and multivariate analyses of 11 variables related to survival of GBC patients with preoperative jaundice (*n* = 54)

Variable	Cutoff level	Number	Survival rates (%)		Univariate	Multivariate	
			1 year	3 years	*P* value	RR (95% CI)	*P*
Age (year)					0.334		
	< 60	26	50.0	11.5			
	≥ 60	28	25.5	15.3			
Sex					0.391		
	Male	30	53.3	6.7			
	Female	24	54.2	20.8			
Preoperative Biliary Drainage					0.151		
	Present	34	51.5	6.1			
	Absent	20	57.1	23.8			
Associated disease					0.672		
	Present	25	44.0	16.0			
	Absent	29	62.1	10.3			
Tumor location					0.050	1.288 (0.626–2.650)	0.492
	Gallbladder neck	38	63.2	15.8			
	Gallbladder body/fundus	16	31.3	6.3			
pT (TNM)					0.010	2.221 (1.065–4.631)	0.033
	pT3	14	64.3	35.7			
	pT4	40	50.0	5.0			
Lymph node metastasis					0.136		
	Negative	15	66.7	13.3			
	Positive	39	48.7	12.8			
Histologic differentiation					0.025	0.521 (0.216–1.253)	0.145
	Well/moderate	47	59.6	14.9			
	Poor	7	14.3	0.0			
CRAO					0.545		
	Present	9	44.4	11.1			
	Absent	45	55.6	13.3			
Intraoperative blood infusion					0.137		
	Present	23	56.5	26.1			
	Absent	31	51.6	3.2			
Morbidity					0.301		
	Present	15	53.3	6.7			
	Absent	39	53.8	15.4			

### Clinicopathologic features of seven 3-year survivors (Table 5)

Of the 54 GBC patients with preoperative jaundice, 7 survived for more than 3 years. They were 2 males and 5 females, with an average age of 57.4 years. Comparing the 7 jaundiced patients who survived for more than 3 years with those who did not, a significant difference of intraoperative blood transfusion and pT stage was observed.

**Table 5:** Compared analyses for jaundiced patients with and without long survival (*n* = 54)

Variables	Survived more than 3 years, *n* = 7	Died within 3 years, *n* = 47	*P* value
Male gender	2	28	0.221
Mean age	57.43 ± 10.96	62.14 ± 10.06	0.288
Associated gallbladder stone	4	21	0.692
Preoperative biliary drainage	2	31	0.096
Tumor location			0.660
Neck	6 (85.7%)	32 (68.1%)	
Body/fundus	1 (14.3%)	15 (31.9%)	
Postoperative hospital stay	12.29 ± 3.82	19.21 ± 13.56	0.188
Bilirubin level at presentation	183.06 ± 103.79	251.93 ± 133.50	0.149
Intraoperative bleeding (ml)	785.71 ± 371.61	637.23 ± 553.49	0.380
Intraoperative blood infusion	6 (85.7%)	17 (36.2%)	0.034
Operative time (min)	320.00 ± 80.83	292.45 ± 76.57	0.423
Combined resection of adjacent organs	1 (14.3%)	8 (17%)	0.670
Hepatic invasion	4 (57.1%)	25 (53.2%)	0.585
pT (TNM)			0.010
pT3	5 (71.4%)	9 (19.1%)	
pT4	2 (28.6%)	38 (80.9%)	
Lymph node metastasis	5 (71.4%)	34 (72.3%)	0.637
Histologic type			0.576
Well + moderately differentiated	7 (100%)	40 (85.1%)	
Poorly differentiated	0 (0%)	7 (14.9%)	
Extent of liver resection			0.219
Major hepatectomy (> 3 segments)	0 (0%)	4 (8.5%)	
Anatomical segments IV–V	1 (14.3%)	19 (40.4%)	
Gallbladder bed	6 (85.7%)	24 (51.1%)	
CRAO			0.670
Yes	1 (14.3%)	8 (17.0%)	
No	6 (85.7%)	3 (83.0%)	
Combined portal vein/hepatic artery resection	0 (0%)	5 (10.6%)	0.485
Incidence of incidental gallbladder cancer (IGC)	1 (14.3%)	1 (2.13%_)	0.2445

## Discussion

This was a large sample study and confirmed that jaundice was a predictor of advanced gallbladder cancer. The 3-year survival rate and median survival time were 13.0% and 18.0 months for the 54 jaundiced patients, respectively, and 40.9% and 40.8 months for the 213 non-jaundiced ones, respectively (*P* < 0.001). The jaundiced patients had significantly lower survival rates than the non-jaundiced patients. In this study, the impact of preoperative jaundice on the prognosis of GBC patients after R0 resection was thoroughly evaluated. So far, no other report is known to be published. This study provided a basis and data support for clinical prognostic evaluation of GBC patients after R0 resection.

In previous reports, preoperative jaundice was an important predictor of advanced gallbladder carcinoma [11]. Although the surgical prognosis of advanced gallbladder cancer was not satisfactory, it was the only expected treatment to be cured [6, 8]. Advanced gallbladder carcinoma was usually accompanied with adjacent organ invasion, such as the liver, transverse colon, duodenum, extrahepatic bile duct, hepatic artery, and portal vein. Enlarged surgical resection was required for radical resection. The morbidity and mortality of this operation were still high. It was reported that the postoperative complication rate was as high as 53% and the mortality was 4–27% in extended hepatectomy [14]. With the advent of preoperative biliary drainage and portal vein embolization (PVE), extended hemihepatectomy, mainly extended right hepatectomy, was safer and more feasible. Although extended hemihepatectomy was expected to remove tumor lesions radically, the postoperative mortality was higher and survival benefits remained controversial [15]. In addition, the local radical resection with more liver parenchyma reservation was expected to achieve similar prognosis [16]. In short, the advantages and disadvantages of extended surgery should be carefully weighed. This study has found that the jaundiced group had a worse prognosis than the non-jaundiced group, suggesting that preoperative jaundice was identified as advanced stage. However, there was no specific correlation between preoperative jaundice and long-term survival.

In previous reports, hilar invasion was identified as an important prognostic factor [10, 17]. The clinical manifestation of hilar invasion was jaundice, which was an independent factor for poor prognosis [10, 17]. In our previous study, the lymph node metastasis and gallbladder neck tumors were the only significant risk factors of poor prognosis in GBC patients who underwent surgical resection with curative intent (R0 and R1 resections) [12]. However, in this study, preoperative jaundice and gallbladder neck tumors were not the independent factors associated with poor prognosis after R0 resection, which suggested that GBC patients with preoperative jaundice and gallbladder neck tumors should be actively given surgery, if R0 resection was expected. This was different from previous research results. This again emphasized the clinical importance of radical resection.

In addition, another important predictor of poor prognosis in gallbladder cancer was lymph node metastasis [17–19]. Kondo et al. [20] insisted that surgery cannot improve the prognosis of gallbladder cancer patients with lymph node metastasis around the abdominal aorta. In contrast, reports from the Japanese Society of Biliary Surgery have found that GBC patients with extensive lymph node metastasis also benefit from lymphadenectomy [10, 19]. The univariate analysis of this study has found that lymph node metastasis was closely associated with survival (*P* < 0.001). However, the multivariate analysis of this study has found that regional lymph node involvement was not an independent prognostic factor for long-term survival, and only pT stage was a key prognostic factor. With the continuous advancement of imaging technologies such as CT, MRI, and PET-CT [21], the pT stage can be more accurately evaluated before surgery. Therefore, once more advanced pT stage (pT4) was suggested by preoperative imaging, the choice of surgical indications and multidisciplinary treatment should be more cautious.

The limitation of this study is the limited amount of cases. Further multicenter studies are needed to confirm this conclusion. In addition, there are limitations in retrospective research itself. In conclusion, this study suggested that there was no absolute relation between preoperative jaundice and poor long-term prognosis. The pT staging was a key long-term prognostic factor for gallbladder carcinoma after R0 resection.

## Data Availability

The datasets used and analyzed during the current study are available from the corresponding author on reasonable request.

## Abbreviation

GBC: Gallbladder cancer,
R0: Radical resection
pT: Depth of tumor invasion
PVE: Portal vein embolization
PTBD: Percutaneous transhepatic biliary puncture
EBD: Endoscopic biliary drainage
CRAO: Combined resection of adjacent organs
PV: Portal vein
HA: Hepatic artery
OS: Overall survival
DFS: Disease-free survival
